# High-throughput detection of potential bacteriocin producers in a large strain library using live fluorescent biosensors

**DOI:** 10.3389/fbioe.2024.1405202

**Published:** 2024-07-31

**Authors:** Sebastian J. Otto, Laura Teichmann, Niklas Fante, Peter Crauwels, Alexander Grünberger, Tobias Neddermann, Christian U. Riedel

**Affiliations:** ^1^ Department of Biology, University of Ulm, Ulm, Germany; ^2^ NovaTaste Production GmbH, Holdorf, Germany; ^3^ Multiscale Bioengineering, Technical Faculty, Bielefeld University, Bielefeld, Germany; ^4^ Microsystems in Bioprocess Engineering, Institute of Process Engineering in Life Sciences, Karlsruhe Institute of Technology (KIT), Karlsruhe, Germany

**Keywords:** bacteriocin, lactic acid bacteria, biosensors, high-throughput screening, fluorescence

## Abstract

The global increase in antibiotic resistances demands for additional efforts to identify novel antimicrobials such as bacteriocins. These antimicrobial peptides of bacterial origin are already used widely in food preservation and promising alternatives for antibiotics in animal feed and some clinical setting. Identification of novel antimicrobials is facilitated by appropriate high throughput screening (HTS) methods. Previously, we have described a rapid, simple and cost-efficient assay based on live biosensor bacteria for detection of antimicrobial compounds that act on membrane integrity using the ratiometric pH-dependent fluorescent protein pHluorin2 (pHin2). Here, we use these biosensors to develop an integrated pipeline for high-throughput identification of bacteriocin producers and their biosynthetic gene clusters. We extend the existing portfolio of biosensors by generating pHin2 expressing strains of *Escherichia coli*, *Bacillus cereus*, *Staphylococcus epidermidis*, and methicillin-resistant *Staphylococcus aureus*. These strains were characterized, and control experiments were performed to assess heterogeneity of these biosensors in response to known bacteriocins and develop a robust HTS system. To allow detection of compounds that inhibit target bacteria by inhibiting growth without disturbing membrane integrity, the HTS system was extended with a growth-dependent readout. Using this HTS system, we screened supernatants of a total of 395 strains of a collection of lactic acid bacteria. After two rounds of screening 19 strains of the collection were identified that produced antimicrobial activity against *Listeria innocua* and *Listeria monocytogenes*. Genomes of confirmed hits were sequenced and annotated. *In silico* analysis revealed that the identified strains encode between one and six biosynthetic gene clusters (BGCs) for bacteriocins. Our results suggest that pHin2 biosensors provides a flexible, cheap, fast, robust and easy to handle HTS system for identification of potential bacteriocins and their BGCs in large strain collections.

## 1 Introduction

Antimicrobial peptides are a host defense mechanism conserved across all domains of life. Bacteriocins are a subset of antimicrobial peptides produced by bacteria and released into the environment to provide a competitive advantage over other bacteria of the same natural habitat. The chemical nature of bacteriocins is diverse and ranges from simple, linear, non-modified peptides to complex structures that are formed from gene-encoded peptides following extensive posttranslational modifications (Sugrue et al., 2024). Although most of the currently known bacteriocins are from lactic acid bacteria (LAB), they are found virtually in any bacterial genus analysed so far (Riley and Wertz, 2002; Li et al., 2021; Lozo et al., 2021). For bacteriocins of Gram-negative bacteria, classification according to size into (i) microcins (<10 kDa), (ii) large colicin-like multidomain proteins (25–80 kDa), and (iii) large phage-like multimeric protein assemblies also termed tailocins has been proposed (Chavan and Riley, 2007). Bacteriocins of Gram-positive bacteria are usually classified by size and physicochemical properties (Darbandi et al., 2022). Class I bacteriocins are small heat-stable peptides with posttranslational modifications (e.g., lantibiotics), class II includes small, heat-stable peptides without unusual modifications, and larger, heat-labile peptides/small proteins are grouped in class III. Recently, a class IV has been added containing bacteriocins with lipid or carbohydrate moieties.

Several mechanisms have been described for bacteriocins including interference with cell wall biosynthesis, enzyme inhibition, and nuclease activity against DNA, rRNA and tRNA (Riley and Wertz, 2002; Yang et al., 2014; Darbandi et al., 2022). However, the most common mechanism by far involves disruption of integrity of the cytoplasmatic membrane by pore formation. Pore-forming bacteriocins usually have an overall positive charge under physiological conditions facilitating attraction and adsorption to the negatively charged cell envelope of targets (Vasilchenko and Valyshev, 2019; Darbandi et al., 2022).

The range of target organisms of bacteriocins largely depends on the receptor and mechanism of pore formation. The receptor for the prototype class I bacteriocin nisin is lipid II (Brötz et al., 1998; Bierbaum and Sahl, 2009), which is an essential precursor of cell wall biosynthesis located in the outer leaflet of the cell membrane. Binding of nisin to lipid II prevents incorporation into the nascent peptidoglycan chain and thus inhibits cell wall biosynthesis and growth. With increasing nisin concentrations, pores are formed in the membrane consisting of nisin and lipid II molecules that cause disruption of membrane integrity and killing of the target (Bierbaum and Sahl, 2009). As lipid II is a highly conserved precursor of cell wall biosynthesis, nisin is active against a wide range of Gram-positive bacteria including clinically relevant and drug resistant pathogens, e.g., enterococci, *Listeria monocytogenes*, (methicillin-resistant) *Staphylococcus aureus*, *Streptococcus pneumoniae*, and *Clostridium difficile* (Shin et al., 2016).

The receptor for all class IIa and some class IId bacteriocins are the IIC and IID subunits of a distinct phylogenetic group of phosphoenolpyruvate-dependent sugar transporters of the mannose family (Man-PTS) that import specific sugars such as mannose, glucose, glucosamine or fructose (Diep et al., 2007; Tymoszewska et al., 2017; Chikindas et al., 2018; Tymoszewska et al., 2018; Desiderato et al., 2022; Vogel et al., 2022). Thus, only bacteria that possess this particular type of Man-PTS are susceptible. For the class IId bacteriocins garvicin A, B and C, Tymoszewska and colleagues have proposed that binding of the peptides to Man-PTS induces conformational changes locking the channel of the transporter in an open position, which results in disruption of membrane integrity (Tymoszewska et al., 2018). This hypothesis was recently confirmed by cryo-electron microscopy for pediocin PA-1 and the Man-PTS of *L. monocytogenes* (Zhu et al., 2022). Structural modelling suggests that the class IId bacteriocin garvicin Q interacts with the Man-PTS of *L. monocytogenes* in a similar fashion (Desiderato et al., 2022).

Due to their antimicrobial activity against important (human) pathogens, bacteriocins are interesting bioactive molecules for a number of applications (Cotter et al., 2005; Chikindas et al., 2018; O'Connor et al., 2020). The most common application of bacteriocins to date is their use in preservation of food and beverages (Cotter et al., 2005; Chikindas et al., 2018; O'Connor et al., 2020). The bacteriocin most widely used in the food industries is nisin, which is approved as a biopreservative in over 80 countries worldwide (Field et al., 2023) and has a projected annual market volume of 553 million USD by 2025 (MarketsAndMarkets, 2023). Currently, nisin and other industrially relevant bacteriocins are marketed either as partially purified compounds, bioactive powders prepared from supernatants of natural bacteriocin producers, or bacteriocin-producing protective cultures (Chikindas et al., 2018). Despite their proven efficacy and safety, there are hardly more than a handful of different bacteriocins available on the market as (semi)purified compounds or bioactive powders (Soltani et al., 2021). However, consumer demand for minimally processed and naturally preserved food products containing bioactive and health-promoting substances is steadily increasing and so is the interest of companies in protective bacterial cultures for fermented food and dairy products (O'Connor et al., 2020; Vinderola et al., 2023).

Recently, we developed a simple assay for detection of bacteriocin activity based on fluorescent sensor bacteria (Crauwels et al., 2018; Reich et al., 2022). These sensor bacteria express pHluorin2 (pHin2), a pH-dependent fluorescent protein with a bimodal excitation spectrum and a ratiometric shift in relative fluorescence intensity at the two excitation peaks in response to changes in pH (Mahon, 2011). Unchallenged sensor bacteria are able to maintain a constant intracellular pH even in acidic buffer systems but when exposed to compounds that disrupt membrane integrity, intracellular pH immediately changes to the extracellular pH of the buffer (Ovchinnikov et al., 2022). This shift can be detected by changes in relative intensity of fluorescence after excitation at 400 and 470 nm. Thus, in an acidic buffer system, bacteria expressing intracellular pHin2 report presence of membrane-damaging compounds within minutes. This facilitates high throughput screening (HTS) of potential bacteriocins in supernatants of collections of bacteria in a rapid and cost-efficient manner (Desiderato et al., 2021).

In the present study, we used this system to establish an integrated pipeline for identification of bacteriocins and the corresponding BGCs in an industrial collection of starter cultures comprising hundreds of strains.

## 2 Materials and methods

### 2.1 Bacterial strains and cultivation conditions

Bacterial biosensor strains *L. monocytogenes* EGD-e/pNZ-pHin2^*Lm*^, *Listeria innocua* LMG2785/pNZ-pHin2^*Lm*^ have been described previously (Reich et al., 2022). New biosensors of *E. coli* MG1655 (Bachmann, 1972), *Bacillus cereus* DSM 31 (German Collection of Microorganisms and Cell Cultures), *Staphylococcus epidermidis* RP62-a (Gill et al., 2005), and methicillin-resistant *S. aureus* Rosenbach (Cowan et al., 1954) were constructed by transformation with the plasmid pNZ-pHin2^*Lm*^ (Reich et al., 2022) using previously described protocols (Bone and Ellar, 1989; Inoue et al., 1990; Monk et al., 2015). Staphylococci are notoriously known for their recalcitrance to standard methods of transformation due to the presence of multiple restriction/modification systems. To overcome this restriction barrier, plasmids must be methylated in the correct pattern using dedicated *Escherichia coli* cloning hosts expressing the right methyltransferases. Plasmids for transformation of staphylococci were thus isolated from *E. coli* IM08B (Monk et al., 2015) for MRSA or *E. coli* Ec_SeRP62aI (Lee et al., 2019) for *S. epidermidis* RP62-a. All biosensors were grown overnight (O/N) in 5–10 mL Brain Heart Infusion broth (BHI) containing 10 μg mL^−1^ chloramphenicol.

The strains of the starter culture collection are listed in Supplementary Data S1 and were grown in MRS medium at 30°C for 20–72 h to stationary growth phase. At this stage, bacteria were pelleted by centrifugation (5,200 × g, 20 min, RT) and supernatants were collected. For all supernatant, pH was neutralized using 1 M NaOH. For the primary HTS, supernatant were roughly adjusted to pH 5.5–6.5 by adding a volume of NaOH estimated based on final OD of the culture assuming acidification correlates at least to a certain degree with OD. Subsequently, pH was briefly verified to be in the range of 5.5–6.5. Only for a few supernatants, pH was outside this range and these outliers were not treated further. This was done to minimize workload for the primary screening. We considered this acceptable as false positive hits due to highly acidic pH picked up in the first HTS were excluded in a secondary screening with supernatants precisely adjusted to pH 6.2, subsequently heat-inactivated (80°C, 15 min) and 1 mL aliquots were stored in deep well plates (Sarstedt, Nümbrecht, Germany) at −20°C until further processing. To characterize the antimicrobial activity in the secondary screening, supernatants were heat inactivated again more thoroughly for some experiments by a second incubation for 15 min at 96°C or treated with proteinase K (0.5 mg mL^−1^, 30 min at 37°C).

### 2.2 Screening procedures

#### 2.2.1 pHin2 assay

Screening for potential bacteriocin producers was performed by analyzing supernatants of the starter collection using pHin2 assays as published previously (Reich et al., 2022) with minor modifications.

Biosensor bacteria were suspended in optimized *Listeria* minimal buffer (LMBO: 200 mM MES, 4.82 mM K_2_HPO_4_, 11.55 mM Na_2_HPO_4_, 1.7 mM MgSO_4_, 0.6 mg mL^−1^ (NH_4_)_2_SO_4_, 55 mM glucose, pH 6.2) to an OD_600_ of 1.5. LMBO has essentially the same composition as previously published LMB (Crauwels et al., 2018) with MES instead of MOPS and pH 6.2 instead of 6.5 resulting in a slightly higher dynamic range, better discrimination of live and dead bacteria in flow cytometry, and improved readouts of samples obtained from supernatants of LAB grown in complex media such as MRS broth.

Assays were performed in standard 96-well microtiter plates (MTPs; Sarstedt) with 50 µL of supernatants loaded into individual wells of the MTP. In the primary HTS, one supernatant was generated for each strain and tested in three technical replicates on different MTPs. For the secondary screen, supernatants of three independent cultivations were prepared per strain and also analyzed in technical triplicates. Each screening plate included several controls. Supernatants of known bacteriocin producers (*L. lactis* B1629 producing nisin Z, *L. sakei* A1609 producing sakacin A; *Pediococcus acidilactici* A1610 producing pediocin PA-1) were used as positive controls. As further controls, nisin (10 μg mL^−1^, Sigma-Aldrich) for disruption of membrane integrity and ampicillin (100 μg mL^−1^) for growth inhibition without damaging membranes were used. As MRSA showed low susceptibility towards nisin, the membrane-disrupting surfactant cetyltrimethylammonium bromide (CTAB) at 0.004% (w/v) was used as positive control in experiments with MRSA. Sterile MRS medium and supernatants of *L. lactis* IL1403, i.e., a strain that does not produce bacteriocins, served as negative controls. After loading MTPs with samples and controls, 50 µL of the biosensor suspension in LMBO was added to each well of the MTP and mixed by orbital shaking for 3 s. MTPs were incubated in the dark for 30 min and fluorescence intensity (FI) of pHin2 was then measured at 520 nm after excitation at 400, 440 and 480 nm using an MTP plate reader (Infinite M200, Tecan, Männedorf, Switzerland).

For determination of the proportion (percentage) of intact bacteria after treatment with supernatants, negative and positive controls included on each screening plate were used for normalization. FI ratio of biosensors treated with sterile MRS was used to define the upper boundary (100% intact bacteria). FI ratios of biosensor bacteria treated with 10 μg mL^−1^ nisin were defined as the lower boundary (0% intact bacteria). The values for each test sample were normalized to the mean value of the untreated controls (set to 100%) and expressed as % intact bacteria. Samples were defined as hits when % intact bacteria were ≤50%.

#### 2.2.2 Growth-dependent assay

For growth-dependent readouts, 100 µL of sterile BHI broth were added to each well of the same HTS plates used for pHin2 assays after fluorescence readings. Plates were then incubated at 37°C under shaking conditions (1,000 rpm, Titramax 100, Heidolph Instruments GmbH and Co. KG, Kelheim, Germany) and absorbance at 600 nm (Abs_600_) was measured in the MTP reader for up to 6 h. For HTS, the change (increase) in Abs_600_ (ΔAbs_600_) between t = 0 and 4 h of incubation was recorded.

To determine the extent of growth inhibition after treatment with supernatants, negative and positive controls included on each screening plate were used for normalization. ΔAbs_600_ of biosensors treated with sterile MRS was used to define the upper boundary (100% growth, no inhibition). ΔAbs_600_ of biosensors treated with 100 μg mL^−1^ ampicillin were subtracted from all other values to define the lower boundary (0% growth, full inhibition). The mean of the readouts for each test sample were normalized to the mean value of the untreated controls (set to 100%) and expressed as % growth. Samples were defined as hits when % growth was ≤50%.

#### 2.2.3 Agar drop test

All supernatants were additionally checked for antimicrobial activity by agar drop assays as described elsewhere (Tichaczek et al., 1992) with minor modifications. Aliquots of a −80°C glycerol stock were spotted using an inoculation loop on BSM agar plates and, after drying, overlayed with 7 mL of a top agar (0.2% glucose and 0.7% agar) containing the respective biosensor strain. After incubation at 30°C for 48 h, agar plates were analyzed for visible zones of inhibition of biosensor growth around the spots of the potential producer organisms.

### 2.3 Viability assay

To assess the effect of acidic pH and lactate on viability of biosensors, *L*. *innocua* LMG2785/pNZ-pHin2^*Lm*^ was harvested from an O/N culture and resuspended to an initial OD_600_ of 1.5 in LMBO at pH 4.0 or pH 6.0 containing 0, 50, or 100 mM lactate. Following incubation for 30 min at room temperature, 10-fold serial dilutions were prepared in LMBO at pH 6 and 20 µL aliquots of appropriate dilutions were spotted to BHI agar plates containing 10 μg mL^−1^ chloramphenicol. Plates were incubated for 48 h and colony forming units (CFU) mL^−1^ were determined.

### 2.4 Whole genome sequencing and bioinformatic analysis

Whole genome sequencing of putative bacteriocin producer strains was performed using Oxford Nanopore Technology (ONT). For preparation of genomic DNA, bacteria were grown O/N in MRS medium. Cells were harvested by centrifugation and high molecular weight genomic DNA was prepared using the MagAttract HMW DNA kit (Qiagen GmbH, Hilden, Germany) following the manufacturer’s protocol for Gram-positive bacteria. Multiplexed sequencing libraries of three (for Flongle flow cells, FLO-FLG001) or 12 samples (for MinION flow cells FLO-MIN106D) were prepared using the rapid barcoding kit (SQK-RBK004). Multiplex libraries were then sequenced on a MinION Mk1B device run by MinKNOW software version 21.10.4. Raw data was base-called using a CUDA-capable RTX 3070 GPU (Nvidia Corporation) in super-accurate mode of the guppy algorithm (v. 6.0.1, ONT). After demultiplexing (guppy-barcoder, ONT), light quality control on raw reads was performed using filtlong v0.2.1 with recommended settings (Wick, 2021). Draft genomes were assembled *de novo* using Flye v2.9 (Kolmogorov et al., 2019) and polished with Medaka v1.7 (ONT). Genome fasta files were used for taxonomic identification on the TYGS server (Meier-Kolthoff and Göker, 2019). Mining for AMP clusters was done using antiSMASH v. 6.0.1 (Blin et al., 2021). Draft genomes were annotated using the NCBI prokaryotic genome annotation pipeline and are publicly available at NCBI (BioProject: PRJNA971182).

### 2.5 HPLC analysis

Lactic acid analysis was performed by high pressure liquid chromatography (HPLC) of supernatants and lactic acid standards using a setup and a method described elsewhere (Schwentner et al., 2021). Briefly, supernatants were analyzed with an Agilent 1,200 series apparatus (Agilent Technologies, Santa Clara, CA, United States) equipped with an organic acid/sugar column and precolumn (organic-acid resin; 300 × 8 mm; CS Chromatographie, Langerwehe, Germany). Isocratic chromatography was realized with a mobile phase of 5 mM H_2_SO_4_ and a constant flow rate of 0.8 mL min^−1^ at 60°C. Signals were recorded with a refractive index detector and the lactate signal was identified in the complex chromatograms obtained with supernatants by comparison to a 50 mM lactate standard solution as external reference.

### 2.6 Microscopic imaging and analysis

Phase contrast and fluorescence images of *L. innocua* LMG2785/pNZ-pHin2^*Lm*^ sensor cells were taken with an inverted fluorescence microscope (Nikon Eclipse Ti2 Series, Nikon GmbH, Germany) with ×100 oil immersion objective (CFI P-Apo DM Lambda ×100 Oil, Nikon). The Sola light engine (SOLA Light Engine, Lumencor Inc., Beaverton, US) was used for fluorescence excitation. Illumination intensity was set to 10%. Two channel fluorescence for the signal readout of pHin2 was accomplished using different filter sets for channel 1 (Excitation 390/40 nm, Emission 520/35 nm) and channel 2 (Excitation 472/30 nm, Emission 520/35 nm). Exposure times were set to 400 ms for channel 1 and 100 ms for channel 2. Imaging was conducted after 30 min incubation of bacteria from an O/N culture in LMBO buffer containing nisin at concentrations ranging from 0 to 5 μg mL^−1^. The Fiji distribution of ImageJ (Schindelin et al., 2012) was used for image analysis. Individual cells were identified and marked manually in phase contrast images. All images analyzed contained <1% non-fluorescent cells, which were excluded from further analysis. Subsequently, FI of marked regions were determined in both fluorescence channels. Additionally, channel specific background signals were obtained and subtracted from mean FI for each pixel. For visualization, the ratio of background-corrected FIs were calculated and assigned a specific value on a blue-to-red false color scale.

### 2.7 Flow cytometry

Flow cytometry of pHin2 biosensor bacteria was performed using an Amnis^®^ CellStream^®^ device equipped with 405 and 488 nm lasers (Luminex Corporation, Austin, US) as described previously (Reich et al., 2022). FlowJo version 10.8.1 (Becton Dickinson) was used for visualization of flow cytometry data and calculation of FI ratios of pHin2 for single cells.

## 3 Results

In previous studies, we developed a simple assay for detection of bacteriocin activity based on *Listeria* spp. biosensors that instantly respond to membrane-damaging compounds by ratiometric changes of a pH-dependent fluorescent protein (Crauwels et al., 2018; Reich et al., 2022). We have also shown that these *Listeria* spp. biosensors allow HTS for bacteriocins in supernatants of collections of bacteria in a rapid and cost-efficient manner (Desiderato et al., 2021). To expand the list of target organisms for HTS we generated further pHin2 sensor strains by introducing the plasmid pNZ-pHin2^*Lm*^ via standard methods into *E. coli* MG1655, *Bacillus cereus* DSM 31, *S. epidermidis* RP62A, and methicillin-resistant *S. aureus* Rosenbach (MRSA).

**FIGURE 1:**
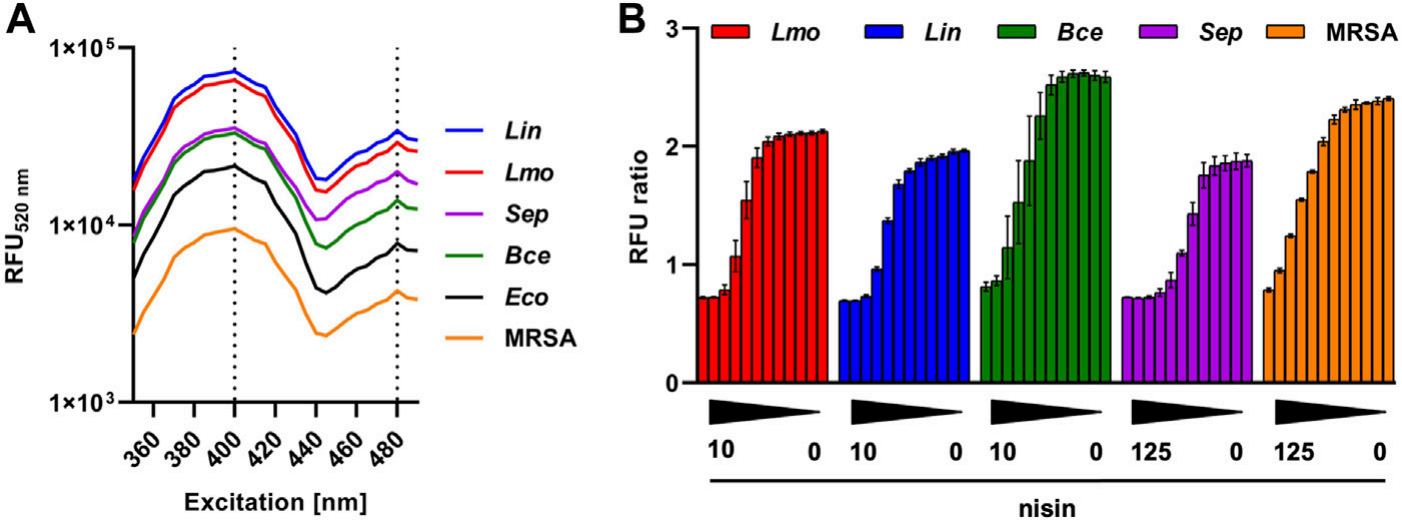
Evaluation of new pHin2 biosensor strains. **(A)** Relative fluorescence units at 520 nm (RFU_520 nm_) of different biosensor strains (*L. monocytogenes*: *Lmo*; *L. innocua*: *Lin*; *E. coli*: *Eco*; *B. cereus*: *Bce*; *S. aureus*: MRSA; *S. epidermidis*: *Sep*) across a spectrum of excitation wavelengths (350–500 nm). Excitation maxima at 400 and 480 nm are highlighted by vertical dashed lines. **(B)** Ratios of pHin2 FI (RFU ratio; emission at 520 nm) with excitation at 400 and 480 nm after treatment of biosensor bacteria with different nisin concentration (decreasing gradient from left to right). Starting concentrations of nisin were 10 μg mL^−1^ (*Lmo*, *Lin*, and *Bce*) or 125 μg mL^−1^ (MRSA and *Sep*). Values are mean ± standard deviation of n = 3 biological replicates (i.e., independent cultivations of biosensors).

All strains exhibited the characteristic excitation maxima of pHin2 at 400 and 480 nm although with minor differences in absolute fluorescence intensity (Figure 1A). To verify functionality of the new biosensors for detection of bacteriocin activity, selected strains were treated with increasing concentrations of nisin and FI ratios of pHin2 after excitation at 400 and 480 nm were determined (Figure 1B). All strains showed a dose-dependent decrease in pHin2 FI ratios. However, nisin concentrations required to achieve a complete drop in pHin2 FI ratio differed slightly between biosensors: 5 μg mL^−1^ for *L. monocytogenes* and *L. innocua*, 10 μg mL^−1^ for *B. cereus*, 31.25 μg mL^−1^ for *S. epidermidis*, and 125 μg mL^−1^ for *S. aureus*. This indicates a markedly higher resistance of staphylococci, and especially the MRSA strain, to nisin compared to the other bacteria investigated.

As observed previously (Crauwels et al., 2018; Reich et al., 2022), the decrease in pHin2 FI ratio is concentration-dependent. For all biosensors tested, a partial decrease of the signal was observed at concentrations lower than those required for complete killing. Similar observations were made for some samples in a screening of supernatants of a small LAB collection (Desiderato et al., 2021). These intermediate signals may either be the result of a homogenous population of biosensors with partially disturbed membrane integrity or a heterogeneous population consisting of dead cells showing fully disrupted membranes and live, intact biosensor. A third possibility is that these intermediate signals are an artefact of the measurement.

**FIGURE 2:**
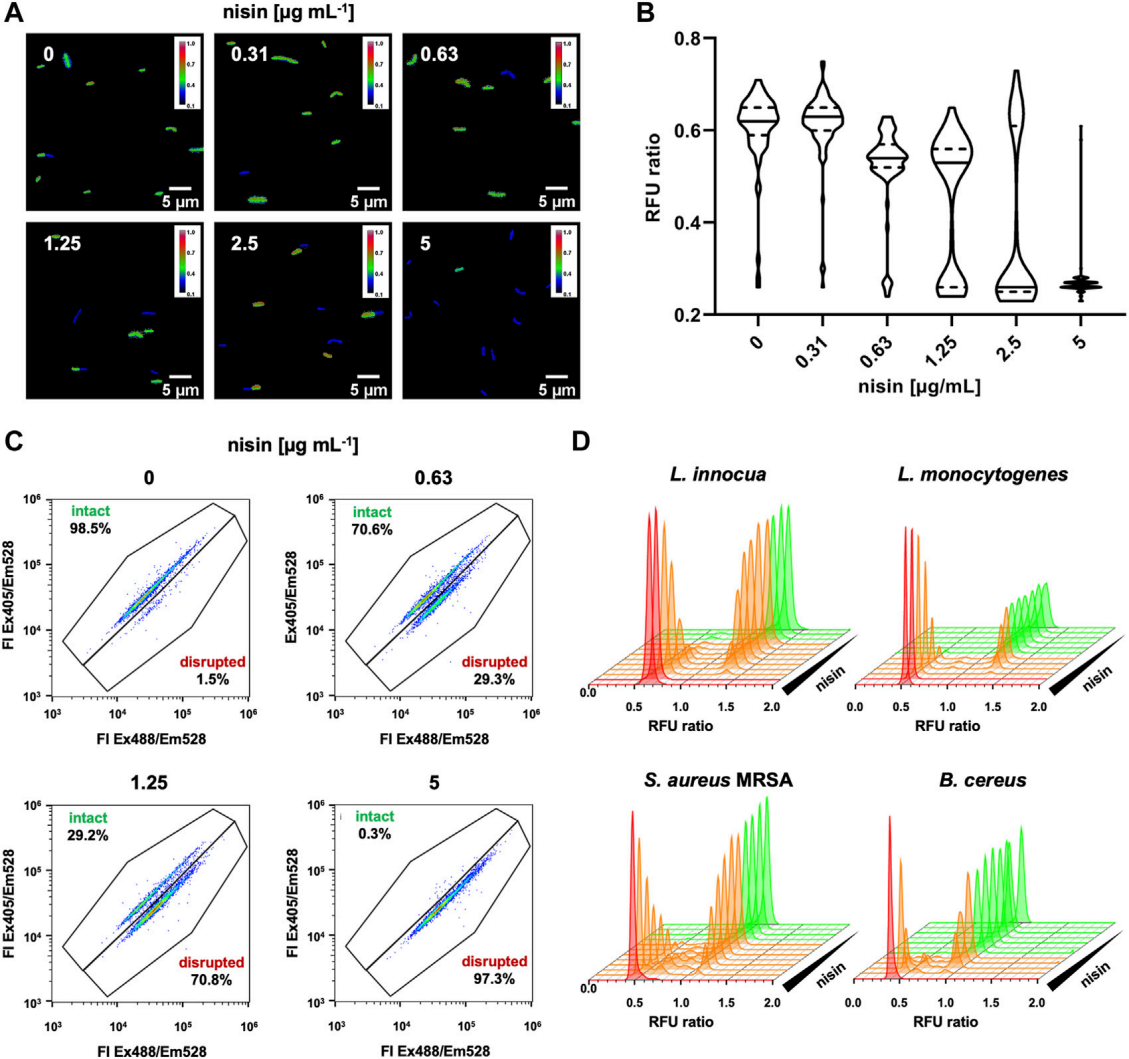
Effects of intermediate nisin concentrations on pHin2 biosensors. **(A)** *L. innocua* LMG2785/pNZ-pHin2^*Lm*^ was treated with nisin at the indicated concentrations and pHin2 relative fluorescence units (RFU) were analyzed via fluorescence microscopy using two different excitation filters with excitation wavelengths of 390 and 472 nm and emission filter at 520 nm. Bacteria were identified in bright field images and RFU in both channels were measured for each pixel. RFU ratios of each pixel were then calculated. Images were generated by plotting the RFU ratio of each pixel on a blue-to-red false color scale. **(B)** Violin plots of quantitative evaluation of mean RFU ratios of 70–120 cells per condition. **(C)** Dot plots analysis of flow cytometry data of approx. 10,000 cells per condition. **(D)** Histogram plots of RFU ratios for single cells of *L. innocua*/pNZ-pHin2^*Lm*^ (upper left panel) using the data shown in C and similar analyses for pHin2 sensor strains *L. monocytogenes*, *S. aureus* MRSA, and *B. cereus*. Nisin concentrations were 2-fold serial dilutions starting with 10 µg mL^−1^ μg mL^−1^ (*Listeria* spp. and *B. cereus*) or 125 μg mL^−1^ (MRSA).

As these questions are of relevance for HTS of larger sample numbers, we further analyzed *L. innocua* LMG2785/pNZ-pHin2^*Lm*^ after treatment with different concentrations of nisin on single cell level by fluorescence microscopy and flow cytometry (Figure 2). Fluorescence microscopy revealed two populations of cells with distinct fluorescence properties (Figure 2A). One population showed higher fluorescence after excitation at 400 nm presumably representing live bacteria with an intact membrane. A second population of (presumably) disrupted, dead bacteria showed higher fluorescence when excited at 472 nm due to membrane damage and drop in intracellular pH. Quantitative images analysis confirmed that both populations are present at intermediate concentrations of nisin. Moreover, the population of live bacteria decreased with increasing concentrations while the dead population increased (Figure 2B).

Analysis of biosensors by flow cytometry confirmed two distinct populations of bacteria that change in relative abundance with increasing nisin concentration (Figures 2C, D). The vast majority (>98%) of untreated *L. innocua* LMG2785/pNZ-pHin2^*Lm*^ biosensors show fluorescence properties of live, intact bacteria. With increasing nisin concentration the proportion of a second population representing disrupted, dead bacteria increases. Following treatment with 5 μg mL^−1^ nisin, membranes of essentially all bacteria (>99%) are disrupted. Similar results were obtained with the other biosensors *L. monocytogenes* EGDe/pNZ-pHin2^*Lm*^, *S. aureus* Rosenbach/pNZ-pHin2^*Lm*^, and *B. cereus* DSM 31/pNZ-pHin2^*Lm*^ (Figure 2D). Overall, this clearly indicates that nisin levels below the minimal bactericidal concentration led to disruption of membrane integrity in some bacteria while others remain unaffected. However, these results also suggest that intermediate signals obtained from non-single cell measurements in microtiter plates can still be considered a valid indicator for presence of compounds that act on membrane integrity, e.g., in a HTS screening for bacteriocins.

**FIGURE 3:**
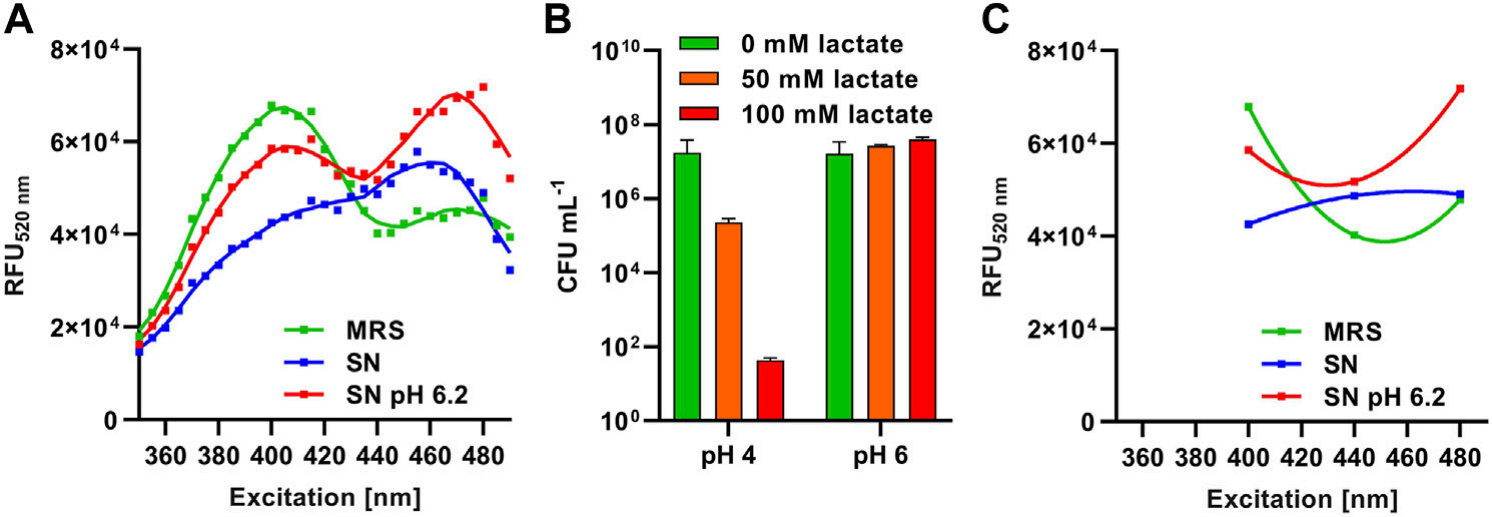
Method development and optimization for HTS procedures. **(A)** Relative fluorescence units at 520 nm (_RFU520 nm_) of *L. innocua*/pNZ-pHin2^*Lm*^ biosensors treated with supernatants of *L. lactis* B1629 before (SN; blue) and after adjustment to pH6.2 (SN pH 6.2; red). Sterile MRS (green) was used as a negative control. **(B)** Viability (CFU mL^-1^) of *L. innocua*/pNZ-pHin2^*Lm*^ incubated with the indicated concentrations of lactate in LMBO at pH four or 6. **(C)** Curves of a second order polynomial regression [f(x) = ax^2^ +bx + c] fitted to the three relative fluorescence intensities of *L. innocua*/pNZ-pHin2^*Lm*^ at 520 nm emission (RFU_520 nm_) after excitation at 400, 440, and 480 nm.

To set up a robust HTS procedure for detection of membrane-damaging activity in complex matrices such as supernatants of bacteria cultivated in MRS broth, further control experiments were performed with the *L. innocua* LMG2785/pNZ-pHin2^*Lm*^ biosensor. The screening protocol was tested with supernatants of *L. sakei* A1609, and *P*. *acidilactici* A1610, and *L. lactis* B1629 known to produce the bacteriocins nisin, sakacin A, or pediocin PA-1, respectively. Use of LMBO allowed detection of bacteriocin activity in plain supernatants [Supplementary Data S2, Supplementary Figure S1 and (Crauwels et al., 2018)]. However, for supernatants of the nisin Z producer *L. lactis* B1629 an unusual FI spectrum of the biosensor was observed with loss of the two distinct peaks and overall lower FI compared to treatment with MRS only (Figure 3A, blue vs. green line). It has been shown that lactate in combination with acidic pH rapidly inactivates *L. innocua* cells in a synergistic manner (Janssen et al., 2007; Miller et al., 2009) and acidification and production of lactic acid are two of the most important criteria for selection of LAB for use in food preservation (Laranjo et al., 2019).

Indeed, supernatants of O/N cultures of *L. lactis* B1629 had a pH of 4–4.5 (data not shown) and approximately 100 mM of lactate were detected by HPLC (Supplementary Data S2, Supplementary Figure S2). We also observed a synergistic bactericidal effect of lactate and acidic pH on *L. innocua* LMG2785/pNZ-pHin2^*Lm*^ (Figure 3B). At pH = 4, already 50 mM of lactate reduced viable counts by approx. two orders of magnitude within 30 min and 100 mM of lactate caused a decrease in viable counts by approx. five logs. By contrast, at pH = 6, the same concentrations of lactate had no effect on viability. In line with these observations, neutralization of the *L. lactis* B1629 supernatant to pH = 6.2 before incubation with *L. innocua* LMG2785/pNZ-pHin2^*Lm*^ restored the normal fluorescence properties of the biosensor and bacteria displayed the typical FI spectrum of bacteria with a disrupted membrane (Figure 3A, red curve).

As acquisition of complete FI spectra between 400 and 480 nm excitation is not compatible with fast and easy measurements of large sample numbers in HTS, we sought for other means to exclude false positive or negative detection due to compromised pHluorin2 signals. The use of FI with excitation at 440 nm as a third measurement allowed discrimination of the typical signals with two peaks from the signal obtained with acidic supernatants containing a high concentration of lactate (Figure 3C). A non-linear regression calculated with a second order polynomial fit (f(x) = ax^2^ +bx + c) using these three datapoints of, e.g., supernatants of *L. lactis* B1629 (pH = 6.2) had a positive ax^2^ component (parabola open to the top) indicating peaks of fluorescence at 400 and 480 nm excitation relative to fluorescence at 440 nm excitation. By contrast, the fit for samples with compromised signals, e.g., supernatants of *L. lactis* B1629 without setting pH to 6.2, had a negative ax^2^ component (parabola open to the bottom). Thus, we included this third FI measurement with excitation at 440 nm as a quality control in all further experiments. During analysis of the HTS data, a second order polynomial fit was calculated using the three FI values and all samples with a negative ax^2^ component were excluded. Furthermore, neutralization of acidic supernatants to approx. 5.5–6.5 was performed by adding a defined volume of NaOH prior to HTS.

**FIGURE 4:**
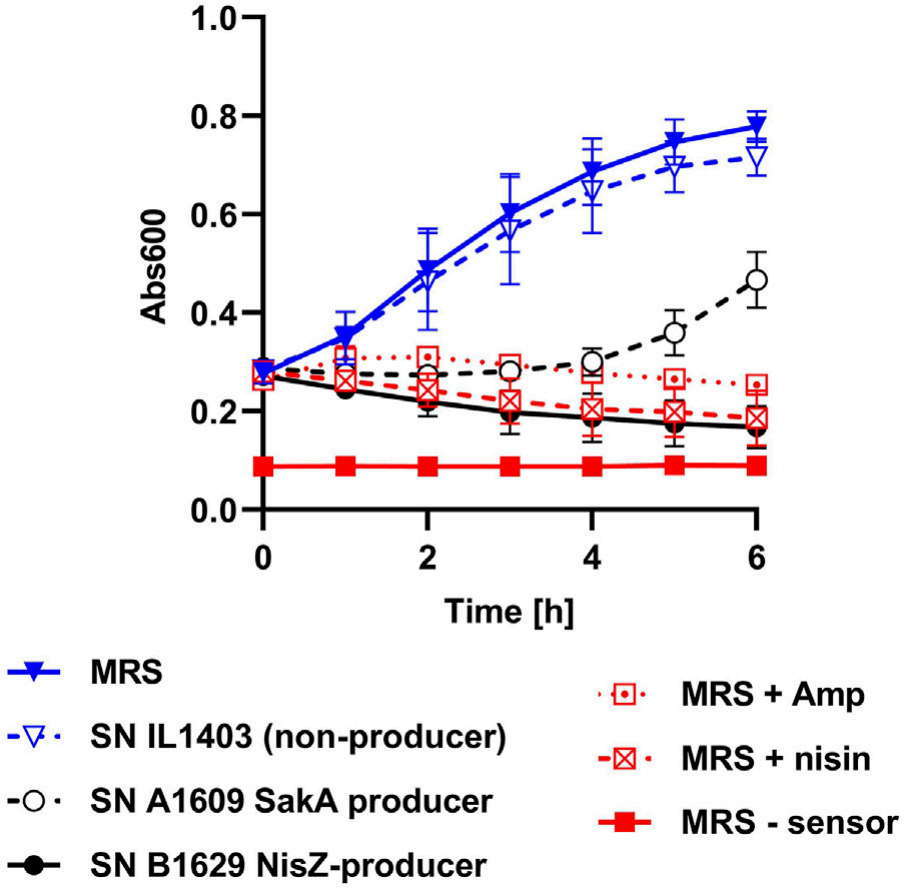
Implementation of a growth readout for HTS. Growth (Abs600) of *L. innocua*/pNZ-pHin2^*Lm*^ in MTP incubated at 37°C under shaking conditions. Each of the wells of the MTP contained 50 µL of biosensor bacteria in LMBO (pH 6.2), 100 µL of BHI and 50 µL of supernatants of either *L. lactis* IL1403, a strain that does not produce a bacteriocin (SN IL1403 non-producer), the sakacin A producer *L. sakei* A1609 (SN A 1609 SakA producer), or the nisin Z producer *L. lactis* B1629 (SN B1629 NisZ producer). As positive controls, MRS containing 100 μg mL^−1^ ampicillin (MRS + Amp), 10 μg mL^−1^ nisin (MRS + nisin) were used as negative control (MRS–sensor). Sterile MRS without antibiotics (MRS) was used as negative control. Values are mean ± standard deviation of n = 3 replicates (i.e., independent cultures of the biosensor).

Based on the results obtained so far, the pHluorin signal of biosensor bacteria was considered a rapid, easy and reliable readout. However, it is limited to compounds that destroy membrane integrity. There are also examples of bacteriocins that act by other mechanisms, e.g., inhibition of growth by interference with DNA replication, transcription, or translation (Riley and Wertz, 2002; Yang et al., 2014; Darbandi et al., 2022). To also allow detection of these bacteriocins, we implemented an additional growth-dependent readout in our screening procedure. This was achieved by adding an additional 100 µL of sterile BHI broth to each well of the MTP and monitoring growth by measuring Abs_600_ over time in the same MTP reader used for the fluorescence measurements (Figure 4).

When testing plain, sterile MRS or supernatant of *L. lactis* IL1304, a strain that does not produce bacteriocins, a marked increase in Abs_600_ was observed after adding BHI medium to biosensors. By contrast, no increase in Abs_600_ was observed when MRS contained either 100 μg mL^−1^ ampicillin or 10 μg mL^−1^ of nisin were used as control samples. Also, growth of the biosensor was efficiently inhibited by supernatant of bacteriocin producers *L. lactis* B1629 (nisin Z) or *L. sakei* 1,609 (sakacin A). Although for the latter a mild increase in Abs_600_ was observed at later timepoints, the results suggest that, under the assay conditions, ΔAbs_600_ is a good indicator of compounds that inhibit growth of the biosensor. Based on these results, t = 4 h was selected as a good compromise between time until readout and discriminatory power of ΔAbs_600_. Thus, ΔAbs_600_ between t = 0 and 4 h was included as a second readout for HTS.

**FIGURE 5:**
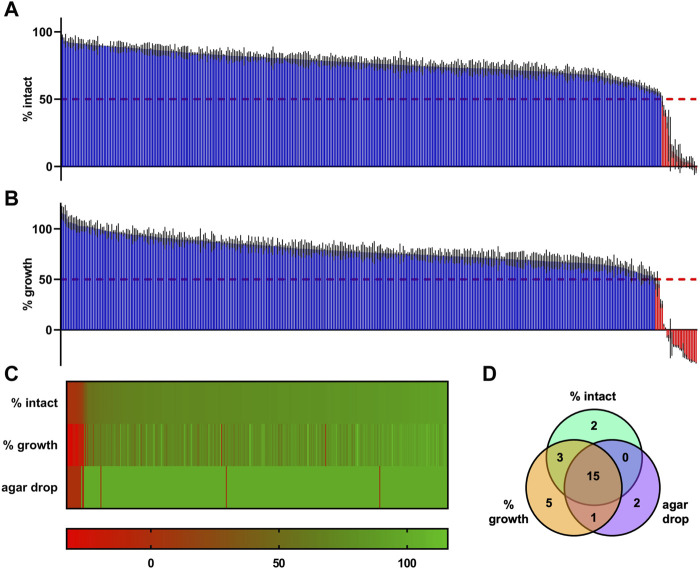
Primary HTS of supernatants of an industrial starter culture collection consisting of 395 strains of lactic acid bacteria. **(A)** Results of the primary HTS using *L. innocua*/pNZ-pHin2^*Lm*^ as biosensor and the pHin2 assay as readout. **(B)** The same MTP plates used for the pHin2 assay were then used for a growth-dependent readout by adding 100 µL of sterile BHI and measurement of Abs_600_ at t = 0 h and t = 4 h of incubation at 37°C with aeration. **(C)** Combined results of HTS readouts in A and B and results of the agar drop HTS by sorting all strains in a heatmap. **(D)** Venn diagram showing the number of hits identified with each HTS and the overlap in hits between the readouts. All values are mean of n = 3 technical replicates for each supernatant (in A, B, and C) with standard deviation (in A and B). Values in A and B are normalized pHin2 FI ratios (expressed as % of intact cells) or normalized ΔAbs_600_ (% growth) with untreated biosensors (neg. controls) set as upper boundary (100%) and nisin-treated (10 μg mL^−1^; pHin2 assay) or ampicillin-treated (100 μg mL^−1^; Abs_600_) set as baseline (0%). Percentages of intact cells or growth were assigned a specific color on a green-to-red color scale. Hits were defined as supernatants that showed a reduction in % intact cells or % growth by at least 50%.

With the optimized setup, we screened supernatants of a collection of 395 starter cultures of LAB including control strains *L. sakei* A1609 and *Pediococcus acidilactici* A1610 for antimicrobial activity against the biosensor *L. innocua*/pNZ-pHin2^*Lm*^ (Figure 5). Using the pHin2 readout, supernatants of 20 strains of the collection (plus the two control strains) passed the threshold, i.e., they showed a reduction in normalized pHin2 FI ratio by at least 50% compared to untreated biosensors (Figure 5A). An additional six strains were identified as potential hits using the growth-dependent readout (ΔAbs_600_; Figure 5B). Of the total of 26 strains, 18 (i.e. 69%) were detected by both assays. To check if antimicrobial activity can also be detected with another method for identification of bacteriocin producers, agar drop assays were performed in which biosensors are in direct contact with bacteriocin producing bacteria instead of their supernatants (Figures 5C, D). Using this more direct method only two further strains showed activity against *L. innocua*/pNZ-pHin2^*Lm*^. Combining the data of all three methods a total of 28 strains plus the two control strains *L. sakei* A1609 and *P*. *acidilactici* A1610 were identified in the collection as potential bacteriocin producers.

**FIGURE 6:**
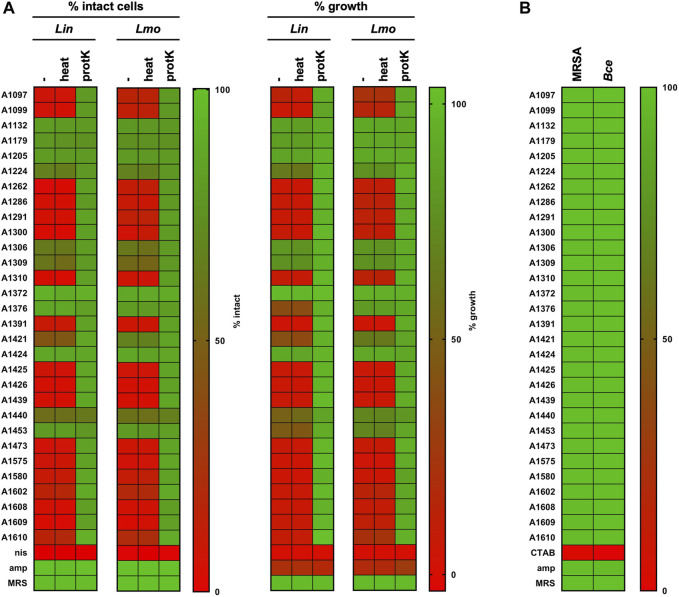
Secondary screen of hits from library. **(A)** Supernatants were pH-adjusted, heat-treated (10 min, 95°C) or incubated with proteinase K (0.5 mg/mL, 30 min, 37°C) and compared to untreated controls using pHluorin and growth-dependent assays with *L. innocua* and *L. monocytogenes* sensors. Nisin (10 μg/mL) and ampicillin (100 μg/mL) were used as controls. **(B)** The same supernatants were also tested against *S. aureus* Rosenbach (MRSA) or *B. cereus* (*Bce*) sensors by flow cytometry using the pHluorin readout. Results of all experiments were normalized as normalized pHin2 FI ratios (expressed as % of intact cells) or normalized ΔAbs_600_ (% growth) with untreated biosensors (neg. controls) set as upper boundary (100%) and nisin-treated (10 μg mL^−1^; pHin2 assay) or ampicillin-treated (100 μg mL^−1^; Abs_600_) set as baseline (0%). Due to the low susceptibility of MRSA towards nisin, the membrane-disrupting surfactant CTAB was used as a control (0.004% w/v). Percentages of intact cells or growth were assigned a specific color on a green-to-red color scale. Hits were defined as supernatants that showed a reduction in % intact cells or % growth by at least 50%. Values are mean of n = 3 of independent cultivations per strain except for MRSA (n = 1).

To exclude false positive hits (e.g., due to insufficient neutralization of pH) a secondary screen was performed for the 28 potential hits and the two control strains (Figure 6). For this secondary screen fresh supernatant were produced, precisely adjusted to pH 6.2 and analyzed with the HTS procedure for pHin2 FI ratio and ΔAbs_600_ (Figure 6A). In the secondary screen, *L. monocytogenes*/pNZ-pHin2^*Lm*^ was used as a second biosensor in addition to *L. innocua*/pNZ-pHin2^*Lm*^. Antimicrobial activity was confirmed for supernatants of 17 strains using the pHin2 readout and supernatants of an additional two strains (A1376 and A1453) showed inhibition of growth. Almost identical results were obtained with both biosensor strains. Additionally, supernatants of the entire collection were also screened for activity using the Gram-negative biosensor *E. coli* MG1655/pNZ-pHin2^*Lm*^ (Supplementary Data S2, Supplementary Figure S3). However, for none of the supernatants activity of >50% reduction in pHin2 FI ratio or growth inhibition was recorded (Supplementary Data S2, Supplementary Figure S4).

To obtain further information on physicochemical properties of the active compounds, supernatants were also tested after treatment with proteinase K or heat (Figure 6A). For all but one supernatants with confirmed antimicrobial activity, protease K treatment completely abolished the activity whereas heat-treatment had no effect. This indicates that the active compounds of these supernatants are indeed heat stable proteins/peptides, which is in line with the definition of class I and II bacteriocins of LAB (Alvarez-Sieiro et al., 2016). The only exception was strain A1440. Supernatants of this strain displayed only very weak activity in the secondary screen that was not affected by proteinase K treatment.

To test if any of the supernatants included in the secondary screen also has antimicrobial activity against bacteria other than *Listeria* spp., they were applied to biosensors of the Gram-positive pathogens *S. aureus* Rosenbach/pNZ-pHin2^*Lm*^ and *B. cereus* 31/pNZ-pHin2^*Lm*^. As for these strains, pHin2 signals were not sufficient for a readout in a MTP reader, analysis was done by flow cytometry. However, no activity against these strains could be observed (Figure 6B).

For identification of biosynthetic gene clusters for bacteriocins, whole genome sequencing was performed for all 19 strains with confirmed antimicrobial activity of supernatants. *De novo* assembly of the read data resulted in draft genomes consisting of 1–15 contigs with sequencing coverage of 39- to 258-fold (data not shown). Draft genomes were subjected to the TYGS server for taxonomic classification (Meier-Kolthoff and Göker, 2019). For identification of RiPP-like antimicrobial peptide gene clusters, genome sequences were analyzed using antiSMASH (Blin et al., 2021) followed by BLAST analysis (Altschul et al., 1990) of the predicted core peptides (Table 1). Of the 19 strains analyzed, 13 belong to the species *Latilactobacillus sakei* and are predicted to produce sakacin A or P. Three strains belong to the species *Pediococccus acidilactici* and are predicted to produce pediocin PA-1. *Lactiplantibacillus plantarum* strains A1376 and A1440 and *Lactiplantibacillus paraplantarum* A1453 harbor genes for the two-peptide bacteriocin plantaricin EF and the two *L. plantarum* strains also encode BGCs for a variety of other peptides with lower similarity to known peptides.

**TABLE 1:** Analysis of whole genome sequencing data. Genome-based identification of bacterial species via TYGS (Meier-Kolthoff and Göker, 2019). Putative encoded core peptides from RiPP-like clusters were predicted using antiSMASH (Blin et al., 2021) and analyzed for identity using BLAST (Altschul et al., 1990).

Strain	Organism	Predicted peptide(s)	% Identity^a^
A1097	*Latilactobacillus sakei*	Bacteriocin sakacin-A	100
A1099	*Latilactobacillus sakei*	Bacteriocin L. sakei^b^	100
		Class IIb bacteriocin, lactobin A/cerein 7B family^b^	100
A1262	*Latilactobacillus sakei*	Bacteriocin sakacin-P	100
A1286	*Latilactobacillus sakei*	Bacteriocin sakacin-A	100
A1291	*Latilactobacillus sakei*	Bacteriocin sakacin-P	100
A1300	*Latilactobacillus sakei*	Bacteriocin sakacin-A	100
A1310	*Latilactobacillus sakei*	Bacteriocin sakacin-A	100
A1376	*Lactiplantibacillus plantarum*	two-peptide bacteriocin plantaricin EF subunits PlnE and PlnF	100, 100
		Hypothetical two-peptide bacteriocin	82, 58
		Brevicin 174A-gamma	100
A1391	*Pediococcus acidilactici*	Bacteriocin pediocin PA-1	100
A1425	*Latilactobacillus sakei*	Bacteriocin sakacin-A	100
A1426	*Latilactobacillus sakei*	Bacteriocin sakacin-P	100
A1439	*Latilactobacillus sakei*	Bacteriocin sakacin-A	100
A1440	*Lactiplantibacillus plantarum*	Brevicin 174A-gamma	100
		Bacteriocin-like peptide^b^	100
		hypothetical protein	64
		bactofencin A family cationic bacteriocin	100
		two-peptide bacteriocin plantaricin EF subunits PlnE and PlnF	100, 100
		Bacteriocin (Lactococcin_972)	100
A1453	*Lactiplantibacillus paraplantarum*	two-peptide bacteriocin plantaricin EF subunits PlnE and PlnF	100, 100
A1473	*Latilactobacillus sakei*	Bacteriocin sakacin-A	100
A1575	*Pediococcus acidilactici*	Bacteriocin pediocin PA-1	100
A1580	*Latilactobacillus sakei*	Bacteriocin sakacin-A	100
A1602	*Pediococcus acidilactici*	bacteriocin transporter in annotation found	
A1608	*Latilactobacillus sakei*	Bacteriocin sakacin-A	100

^a^ Identity of core peptide amino acid sequence to closest hit in BLAST, analysis.

^b^ Not predicted by antiSMASH, but found in bakta annotation.

## 4 Discussion

In face of the dramatic increase in antibiotic resistances of a wide range of bacteria alternative treatment options for infections with these organisms are urgently needed (WHO, 2017). Due to their antimicrobial activity, bacteriocins have potential in applications beyond their traditional use in food preservation. As some bacteriocins are active against important human and antibiotic-resistant bacterial pathogens, they may replace conventional antibiotics in some clinical settings, e.g., for the topical treatment of wounds, but also in animal and pet feed (Cotter et al., 2005; Chikindas et al., 2018; O'Connor et al., 2020). To accelerate identification of novel bacteriocins and their production and their development into (clinical) applications appropriate high throughput screening methods are required. In the present study, we develop an integrated pipeline for high-throughput identification of bacteriocin producers and their biosynthetic gene clusters.

Recently, we developed a simple assay that can be used for detection of bacteriocin activity using sensor bacteria expressing the ratiometric pH-dependent fluorescent proteins pHluorin/pHluorin2 (Crauwels et al., 2018; Reich et al., 2022). These sensor bacteria report presence of membrane-damaging compounds within minutes and facilitate high throughput screening (HTS) of potential bacteriocins in supernatants of collections of bacteria in a rapid and cost-efficient manner (Desiderato et al., 2021). To expand the flexibility of the assay for HTS, we aimed at generating further pHin2 biosensors. The first pHin2 biosensors were strains of *L. monocytogenes* and *L. innocua* (Crauwels et al., 2018; Reich et al., 2022) using the pNZ44 vector backbone (McGrath et al., 2001) with the pSH71 replicon (De Vos, 1987). Initially constructed as a high-copy *E. coli*/*L. lactis* shuttle vector, this replicon is compatible with a wide range of Gram-positive species. Consequently, pNZ-pHin2^*Lm*^ was successfully used to generate a *L. lactis* biosensor to investigate activity and recombinant production of garvicin Q (Desiderato et al., 2022). Here, we use the same plasmid for construction of pHin2 biosensor strains for *E. coli*, *S. aureus*, *S. epidermidis* and *B. cereus* (Figure 1). Although some of these biosensors showed somewhat lower fluorescence intensity than, e.g., *L. innocua*/pNZ-pHin2^*Lm*^ (data not shown) they displayed the characteristic bimodal excitation spectrum and were suitable to detect activity of bacteriocins in pHin2 assays (Figure 1). This highlights the flexibility of the system. Moreover, the setup can be easily adapted by changing the plasmid backbone and/or codon optimization of the pHin2 gene as shown recently by generation of a *Corynebacterium glutamicum* biosensor harboring pPB-pHin^*Cg*^ (Weixler et al., 2022).

To setup a robust screening system, we performed a number of control experiments. First, we addressed the source of the intermediate signals (reduction in pHin2 FI ratio) of biosensors treated with sub-MIC concentrations of nisin and analyzed in a MTP reader (Figure 1B). This has been observed for all biosensors reported so far (Crauwels et al., 2018; Desiderato et al., 2022; Reich et al., 2022; Weixler et al., 2022; Desiderato et al., 2023). However, it was unclear if this is the result of a homogenous population of cells with impaired pH homeostasis of the integrated signals of distinct subpopulations. Extensive analysis of *L. innocua*, *L. monocytogenes*, *S. aureus* and *B. cereus* by both fluorescence microscopy and flow cytometry on single cell level show that sub-MIC concentrations of nisin clearly result in two distinct populations of (i) live, intact cells and (ii) cells with a completely disrupted membrane. We did not observe a third population of sublethally stressed bacteria previously shown, e.g., by flow cytometric analysis of *L. monocytogenes* treated with herbal essential oils (Paparella et al., 2008).

It has been shown that the activity of nisin is mediated via lipid II, an essential precursor of cell wall biosynthesis and subsequent formation of pores in the membrane (Breukink and de Kruijff, 2006). A possible explanation for the presence of two distinct populations is that for those bacteria that are killed at sub-MIC nisin levels the threshold concentration for pore formation at the membrane is reached whereas for those that remain unaffected it is not. Whether this is caused by differences in the composition of the cell envelope (i.e., in some bacteria nisin reaches the membrane more efficiently than in other) or a scavenging effect (i.e., some bacteria bind nisin more efficiently reducing available nisin in the environment) remains to be elucidated by further studies. Some of these aspects, e.g., time-dependent heterogeneity of *L. innocua*/pNZ-pHin2^*Lm*^ in response to exposure to various nisin concentrations, have been recently been studied in more detail (Fante et al., 2024).

Regarding the goal to establish a robust HTS system, our results demonstrate that even intermediate signals in the pHin2 readout are indicative of presence of a membrane-damaging compound. We were thus confident to set the threshold for defining hits in our HTS at 50% reduction in pHin2 FI ratio. Robustness of the screening system was further increased by including a third “quality control” fluorescence measurement at 440 nm excitation and calculation of a second order polynomial fit. This excludes false positive interpretation of the pHin2 FI ratio, e.g., due to presence of lactate and acidic pH. The vast majority of supernatants that were not confirmed in the secondary screen were only identified by one of the three readouts in the primary screen and/or had very low activity. As the only parameter changed for the secondary screen was precise adjustment of pH, this suggests results of these supernatants were indeed biased by antimicrobial effects of acidic pH and lactate and were thus false positives.

One major advantage of screening for antimicrobial activity using pHin2 biosensors over classical, growth-based assays (e.g., spot-on-lawn or well/disk diffusion assays, 96-well growth assays, etc.) is the a dramatically reduced time until detection. With growth dependent approaches, it easily takes 24–48 h to obtain results depending on the indicator organism (Balouiri et al., 2016). Approaches with shorter assay times are, e.g., ATP bioluminescence assay or propidium iodide staining, but they require addition of enzymes, substrates (luciferase/luciferin), or dyes and, in some cases, expensive equipment (flow cytometry) for detection (Balouiri et al., 2016). The assay based on pHin2 biosensors provides fast and reliable readouts within 2 h including preparation of biosensors and sample, fluorescence measurements are easy to implement on a variety of HTS-compatible technologies (MTP, microscopy, flow cytometry) without the need for addition of exogenous substances. However, pHin2 biosensor assays are limited to detection of antimicrobial substances that act on membrane integrity. Antimicrobial compounds including bacteriocins that act by inhibition of growth via other mechanisms (Riley and Wertz, 2002; Yang et al., 2014; Darbandi et al., 2022) are not detected by the pHin2 readout. To allow detection of such compounds, we thus implemented an additional growth dependent readout for the same assay plates. Only two supernatants of the collection (strains 1,376 and 1,453) consistently showed growth inhibition but not pore formation. Nevertheless, these results demonstrate that both readouts yield distinct hits.

Bacteriocins are a widely used mechanism of competition providing an advantage in the respective ecological niche or habitat to the producer strains. BGCs were found in virtually any bacterial genus analyzed and 30%–99% of bacterial species are estimated to encode at least one BGC (Riley and Wertz, 2002). For example, roughly 25% of the *Corynebacterium* spp. and *Lactobacillus* spp. genomes (Collins et al., 2017; Goldbeck et al., 2021) and 75% of *Geobacillus* spp. (Egan et al., 2018) contain putative BGCs. Similarly, 59 of the 382 (15%) reference genomes of gastrointestinal bacteria of the human microbiome project harbor at least one BGC (Walsh et al., 2015). In these studies BCG identification was achieved by analysis of genome sequence using standard bioinformatic tools such as BAGEL4 (Van Heel et al., 2018) or antiSMASH (Blin et al., 2021) that basically identify BGCs by similarity to already known bacteriocins. Thus, the total number bacteriocins in nature and our collection may actually be even higher than currently anticipated.

After two rounds of screening, antimicrobial activity was found in supernatants of a total of 19 strains of our collection and activity of the hits of our library was limited to *L. innocua* and *L. monocytogenes* (Figure 6). A complete primary HTS of all supernatants using an *E. coli* biosensor as well as testing the hit supernatants with *B. cereus* and *S. aureus* MRSA biosensors did not identify further activities. This argues for a classical “you get what you screen for” dilemma. LAB usually do not produce colicins or other bacteriocins active against Gram-negative bacteria such as *E. coli*. The collection screened consists of *Latilactobacillus* spp., *Lactiplantibacillus* spp., and *Pediococcus* spp. strains known to produce pediocin-like bacteriocins active against *Listeria* spp. In line with this, corynardin was identified by *in silico* analysis of a *Corynebacterium lactis* genome and is active mostly against other corynebacteria (Pashou et al., 2023) and cutimycin, a thiopeptide bacteriocin with activity against skin pathogens *S. aureus* and *S. epidermidis*, was identified in the skin commensal *Cutibacterium acnes* (Claesen et al., 2020). Thus, it seems reasonable to suggest screening of more closely related bacteria or bacteria from the same habitat will allow identification of producers of bacteriocins active against *E. coli* or *S. aureus*.

21 positive hits (supernatants of 19 newly identified strains plus two controls) in a collection of 395 strains corresponds to roughly 5%. Compared to the genome-mining based estimation that about 25% of *Lactobacillus* spp. genomes contain BGCs (Collins et al., 2017), this number is rather low. However, it has to be taken into consideration that production of bacteriocins may be subject to regulation by conditions of growth, environmental stimuli and/or presence of the targets. For example, some bacteriocins are (auto)regulated by two- or three-component systems (Arbulu and Kjos, 2024). More recently, it was shown that levels of cutimycin in supernatants of *C*. *acnes* is enhanced in the presence of its targets *S. aureus* and *S. epidermidis* (Claesen et al., 2020). This highlights the need for appropriate conditions of cultivation when using activity-based screening methods. In our case, the collection was composed of strains mostly isolated from fermented food. Thus, screening supernatants obtained from cultures grown under conditions closely mimic the natural environment might actually yield more hits. However, identification of bacteriocins by activity-based assays is not possible when target organisms and mode of action are unknown. On the other hand, genome mining is only possible for bacteria with known genome sequences and will only yield BGCs with similarity to known bacteriocins. Thus, activity-based assays for HTS and genome mining approaches have complementary applications and have their role in identification of novel antimicrobials.

Overall, we provide a robust pipeline for HTS for identification of antimicrobial compounds and their BGCs. The HTS system is based on two different readouts that provide information on compounds with activity against live biosensors either by affecting membrane integrity or inhibiting growth by other mechanisms. It is easy to use, provides fast readouts, does not require expensive equipment, additional substrates or dyes and can easily be transferred to other bacteria. Thus, it is highly flexible and can be used for identification of producers of antimicrobial compounds in large collections of microorganisms from a wide range of habitats.

## Data Availability

The dataset of the bacterial genomes sequenced in this study are available online in the BioProject database of the National Center for Biotechnology Information (https://www.ncbi.nlm.nih.gov/bioproject/PRJNA971182).
