# Supplementary material for: High-throughput detection of potential bacteriocin producers in a large strain library using live fluorescent biosensors
Source: Front Bioeng Biotechnol. 2024 Jul 31;12:1405202. doi: 10.3389/fbioe.2024.1405202 (PMC11321961; doi:10.3389/fbioe.2024.1405202)
Supplement: Supplementary file 1 [file DataSheet1.pdf]

Supplementary Material

High-throughput detection of potential bacteriocin producers in a large strain library using live fluorescent biosensors

Sebastian J. Otto[1], Laura Teichmann[2], Niklas Fante[3], Peter Crauwels[1], Alexander Grünberger[3,4], Tobias Neddermann[2], and Christian U. Riedel[1,*]

[1] Department of Biology, University of Ulm, Ulm, Germany

[2] NovaTaste Production GmbH, Holdorf, Germany

[3] Multiscale Bioengineering, Technical Faculty, Bielefeld University, Bielefeld, Germany

[4] Microsystems in Bioprocess Engineering, Institute of Process Engineering in Life Sciences, Karlsruhe Institute of Technology (KIT), Karlsruhe, Germany

[*] **Correspondence:** christian.riedel@uni-ulm.de; ORCID: 0000-0001-7134-7085

1 Supplementary Figures

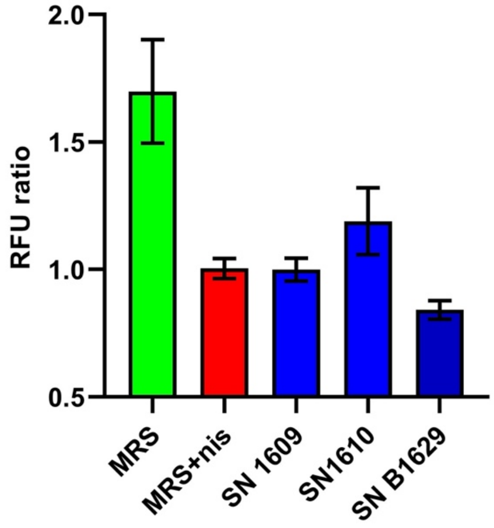

Figure S1: pHin2 assays with supernatants of different control strain using *L. innocua* LMG2785/pNZ-pHin2^{Lm}. biosensors. *L. innocua* LMG2785/pNZ-pHin2^{Lm} was resuspended in LMBO and incubated with supernatants (SN) of *L. sakei* A1609, *Pediococcus acidilactici* A1610, or *L. lactis* B1629 obtained after overnight growth in MRS medium. Sterile MRS with or without nisin (10 µg mL^{-1}) used as controls. Bacteria were then analyzed for pHin2 fluorescence and results are expressed as ratios of fluorescence intensity (RFU ratio, emission at 520 nm) after excitation at 400 and 480. Values are mean ± standard deviation of n = 2-3 replicates (i.e. independent cultures of the biosensor).

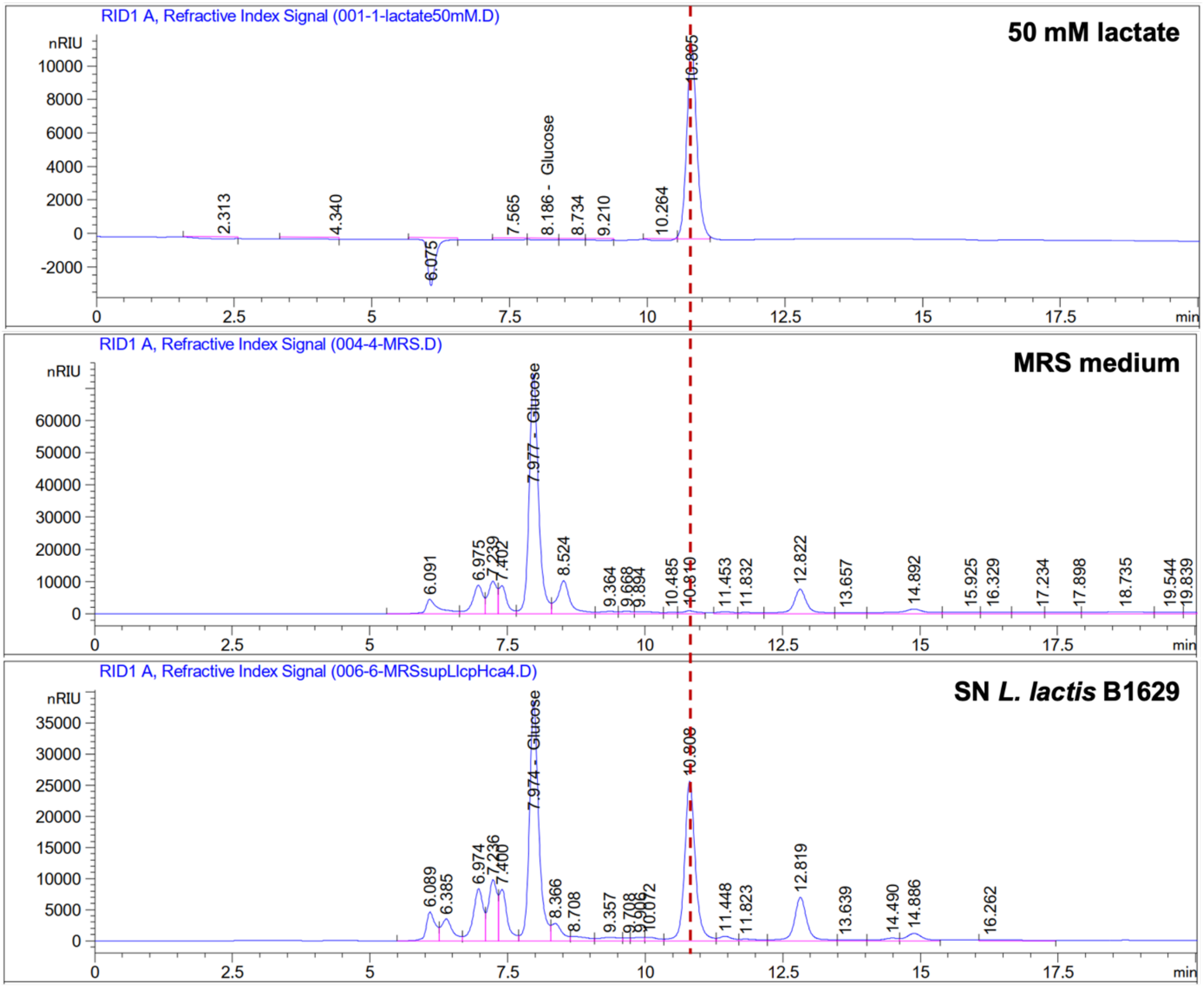

Figure S2: HPLC analysis confirms presence of lactate in supernatants of *L. lactis* B1629. Supernatants (SN) were obtained after cultivation for 16 h in MRS medium. As controls pure lactate (50 mM in H_2O) or sterile MRS medium were analyzed. HPLC was performed on a Agilent 1200 series apparatus (Agilent Technologies, Santa Clara, CA, United States) with a Refractive Index Signal Detector (RID) and signals were recorded as normalized refractory index units (nRIU). The dashed vertical line indicates the peaks for lactate at t = 10.8 min of retention. Lactate concentrations in SN was estimated to be >100 mM based on peak heights (25 000 nRIU for SN vs. 10 000 nRIU for 50 mM lactate standard).

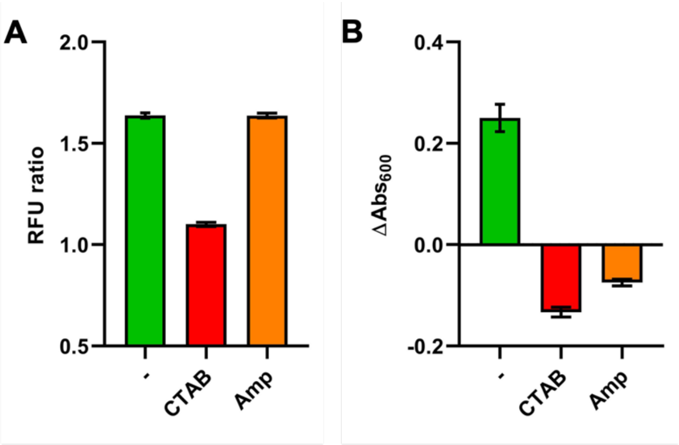

Figure S3: Validation of pHin2- and growth-dependent readouts of *E. coli* MG1655/pNZ-pHin2^{Lm}. *E. coli* MG1655/pNZ-pHin2^{Lm} was resuspended in LMBO and treatment with either CTAB to disrupt membrane integrity or ampicillin (100 µg mL^{-1}) to inhibit growth without membrane damage. (A) Bacteria were then analyzed for pHin2 fluorescence and results are expressed as ratios of fluorescence intensity (RFU ratio, emission at 520 nm) after excitation at 400 and 480. (**B**) The same MTP plates were then used for a growth-dependent readout by adding 100 µl of sterile BHI and measurement of Abs_{600} at t = 0 h and t = 4 h of incubation at 37˘ °C with aeration. Results are shown as changes in Abs_{600} between the two measurements (ΔAbs_{600}). All values are mean ± standard deviation of n = 3 replicates (i.e. independent cultures of the biosensor).

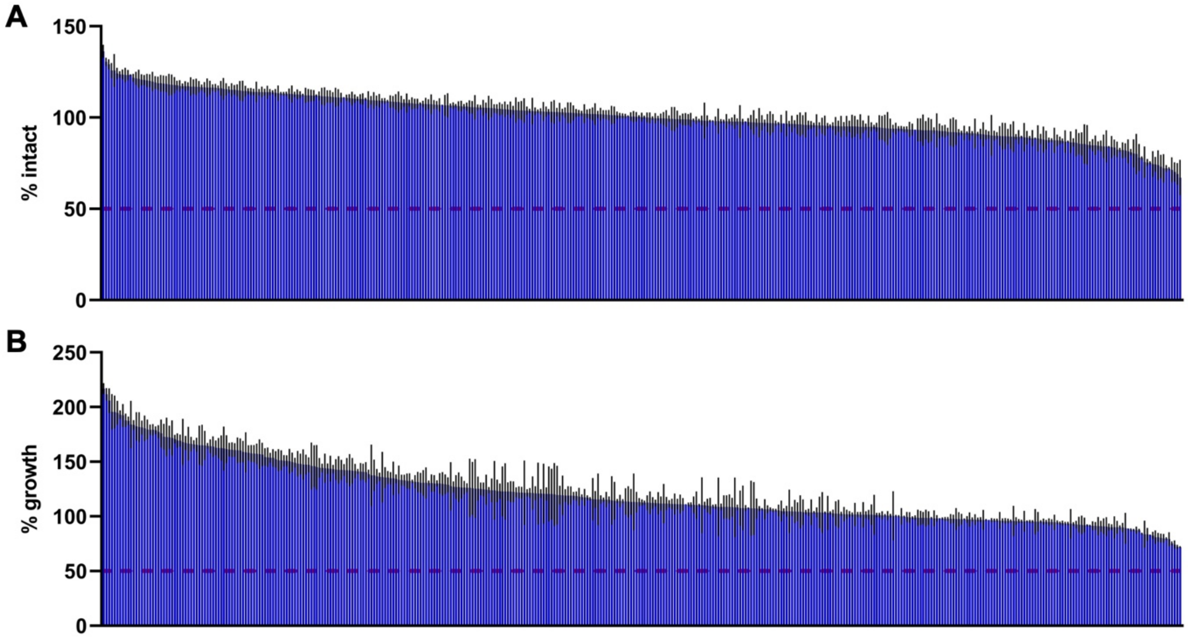

Figure S4: HTS of the supernatant library against the Gram-negative sensor strain *E. coli* MG1655/pNZ-pHin2^{Lm}. (**A**) Results of the pHin2 assay as readout. (**B**) The same MTP plates analyzed for pHin2 FI ratios assay were then used for a growth-dependent readout by adding 100 µl of sterile BHI and measurement of OD_{600} at t = 0 h and t = 4 h of incubation at 37 °C with aeration. All values are mean of n = 3 technical replicates for each supernatant with standard deviation. Values were normalized pHluorin ratios (expressed as % of intact cells) or normalized ΔAbs_{600} (% growth) with untreated biosensors (neg. controls) set as upper boundary (100%) and nisin-treated (10 µg mL^{-1}; pHin2 assay) or ampicillin-treated (100 µg mL^{-1}; Abs_{600}) set as baseline (0%).
